# Niacinamide modulation by hemocyanin shapes hemolymph microbial communities in Penaeid shrimp

**DOI:** 10.1093/ismeco/ycag119

**Published:** 2026-05-07

**Authors:** Shiyuan Bao, Mingming Jiang, Jude Juventus Aweya, Yongsheng Zhang, Xianliang Zhao, Yongzhen Zhao, Chunling Yang, Shengkang Li, Zhihong Zheng, Yueling Zhang

**Affiliations:** Guangdong Provincial Key Laboratory of Marine Biotechnology, Institute of Marine Sciences, Shantou University, Shantou, Guangdong 515063, China; Key Laboratory of Tropical Marine Ecosystem and Bioresource, Fourth Institute of Oceanography, Ministry of Natural Resources, Beihai, Guangxi, 536000, China; Guangdong Provincial Key Laboratory of Marine Biotechnology, Institute of Marine Sciences, Shantou University, Shantou, Guangdong 515063, China; Guangdong Provincial Key Laboratory of Marine Biotechnology, Institute of Marine Sciences, Shantou University, Shantou, Guangdong 515063, China; Department of Food and Human Nutritional Sciences, University of Manitoba, Winnipeg, MB, R2H 2A6, Canada; The Canadian Centre for Agri-Food Research in Health and Medicine, St Boniface Hospital Albrechtsen Research Centre, Winnipeg, MB, R2H 2A6, Canada; Key Laboratory of Tropical Marine Ecosystem and Bioresource, Fourth Institute of Oceanography, Ministry of Natural Resources, Beihai, Guangxi, 536000, China; Guangdong Provincial Key Laboratory of Marine Biotechnology, Institute of Marine Sciences, Shantou University, Shantou, Guangdong 515063, China; Guangxi Academy of Fishery Sciences, Guangxi Key Laboratory of Aquatic Genetic Breeding and Healthy Aquaculture, Nanning, Guangxi 530021, China; Guangxi Academy of Fishery Sciences, Guangxi Key Laboratory of Aquatic Genetic Breeding and Healthy Aquaculture, Nanning, Guangxi 530021, China; Guangdong Provincial Key Laboratory of Marine Biotechnology, Institute of Marine Sciences, Shantou University, Shantou, Guangdong 515063, China; Guangdong Provincial Key Laboratory of Marine Biotechnology, Institute of Marine Sciences, Shantou University, Shantou, Guangdong 515063, China; Guangdong Provincial Key Laboratory of Marine Biotechnology, Institute of Marine Sciences, Shantou University, Shantou, Guangdong 515063, China

**Keywords:** hemolymph microbial homeostasis, niacinamide, respiratory proteins, hemocyanin, shrimp

## Abstract

In aquatic invertebrates, hemolymph is critical for host–environment interaction. Although maintaining microbial homeostasis in hemolymph is significant for disease pathogenesis, the key regulatory mechanisms remain elusive. This study investigates the essential role of the respiratory glycoprotein hemocyanin (HMC) in modulating hemolymph microbial composition in penaeid shrimp. Hemocyanin-depleted shrimp infected with *Vibrio parahaemolyticus* (Vp) exhibited disruptions in the niacinamide (NAM) salvage pathway, attenuated plasma niacinamide and nicotinamide adenine dinucleotide (NAD) levels, and reduced transcripts of key enzymes. These alterations correlated with an increased total bacterial load and a higher abundance of opportunistic pathogens like *Vibrio* and *Shewanella*. Hemocyanin likely modulates niacinamide metabolism through interactions with *Litopenaeus vannamei* nicotinamide riboside kinase 1 (*Lv*NRK1), sirtuin 2 (*Lv*SIRT2), and sirtuin 6 (*Lv*SIRT6). Remarkably, niacinamide supplementation after hemocyanin knockdown restored hemolymph microbial balance, reduced *Vibrio* dominance, and improved shrimp survival against *V. parahaemolyticus* infection, but not Gram-positive *Staphylococcus aureus* (Sa). These findings provide novel insights into the pivotal role of niacinamide metabolism in regulating shrimp hemolymph microbial composition via hemocyanin.

## Introduction

Most animals harbor diverse microbial communities that contribute to nutrition, immune defense, and disease control [1–3]. Host-associated microbiota forms a dynamic microenvironment shaped by long-term interactions between the host and environmental microorganisms [4, 5]. In vertebrates, microbiota mainly colonize body surfaces and cavities connected to the external environment, such as the skin and gut [6]. Although blood was traditionally regarded as sterile [7], the discovery of blood microbiota and circulating microbial metabolites has expanded our understanding of host–microbe interactions in internal body compartments [8]. These circulating microbes and metabolites have been implicated in immune surveillance and a range of inflammatory and metabolic disorders [9, 10].

Aquatic environments harbor highly diverse and dynamic bacterial communities [11]. Therefore, in aquatic organisms with a semi-open circulatory system, hemolymph represents a critical host–environment interface and must maintain microbial homeostasis despite continuous exposure to environmental microorganisms [12]. In mollusks and crustaceans, many hemolymph-associated bacteria are opportunistic pathogens, yet under normal conditions they are maintained in a relatively stable state rather than causing septic disease [13–15]. As the main circulatory fluid in invertebrates, hemolymph and its microbiota are thus crucial for host immunity and disease susceptibility [16]. In aquatic invertebrates, hemolymph bacteria are largely derived from the environment [17]. Because these animals lack adaptive immunity mediated by antibodies and T/B cells, they rely mainly on innate immune mechanisms, including humoral responses, phagocytosis, encapsulation, and nodulation [18, 19]. Accordingly, the hemolymph contains numerous antibacterial effectors, including antimicrobial peptides, lectins, and other active molecules that influence microbial composition [20]. This controlled interaction with resident microbes may also contribute to immune preparedness against invading pathogens [21].

Niacinamide (NAM), an amide derivative of vitamin B_3_, is a key precursor of nicotinamide adenine dinucleotide (NAD) and nicotinamide adenine dinucleotide phosphate (NADP) and is essential for cellular energy metabolism and homeostasis [22–24]. As an important substrate cycle intermediate, NAD supports the activity of enzymes such as sirtuins, poly (ADP-ribose) polymerases (PARP), and CD38, thereby linking niacinamide metabolism to multiple aspects of cellular regulation [25, 26]. In addition to its metabolic role, niacinamide has important immunomodulatory functions. It can inhibit biofilm formation of *Propionibacterium acnes* and suppress *P. acnes*–induced IL-8 production in keratinocytes through the nuclear factor kappa B (NF-κB) and mitogen-activated protein kinase (MAPK) pathways [27]. Niacinamide also replenishes NAD and adenosine triphosphate (ATP); protects cells against oxidative stress, apoptosis, and necrosis; and enhances antimicrobial peptide expression in intestinal epithelial cells [28–30]. However, whether niacinamide metabolism contributes to hemolymph microbiota homeostasis in aquatic invertebrates remains unknown.

Penaeid shrimp, particularly the Pacific white shrimp (*Litopenaeus vannamei*), is the most widely farmed aquaculture species worldwide, yet bacterial diseases such as *Vibrio*-associated acute hepatopancreatic necrosis disease (AHPND) have caused major economic losses [31]. Disease onset in shrimp is often accompanied by microbial dysbiosis, especially enrichment of pathogenic *Vibrio* species [32–34]. As an intermediary between the host and its semi-open environment, hemolymph plays an important role in maintaining microbial homeostasis and thereby influences shrimp health and immunity [15]. Recent studies have shown that several hemolymph components, including C-type lectins and FOXO, actively regulate hemolymph microbial homeostasis [20, 35, 36]. Hemocyanin (HMC), the major respiratory protein in arthropods and mollusks, accounts for >90% of the soluble proteins in shrimp hemolymph [37] and is increasingly recognized as a multifunctional immune-related factor [38], including roles in shaping microbial composition in the shrimp hepatopancreas [39]. Therefore, given its abundance and immune relevance in hemolymph, we asked whether hemocyanin regulates hemolymph microbiota. Here, we show that during *Vibrio parahaemolyticus* (Vp) infection, hemolymph hemocyanin modulates niacinamide levels to shape microbial composition and distribution. These findings provide new insight into how host factors coordinate immunometabolism and microbial homeostasis in penaeid shrimp.

## Materials and methods

### Experimental animals, bacteria, and ethics

Healthy and diseased Pacific white shrimp (*L. vannamei*; 7 ± 0.5 g) were obtained from Fuzeng Aquaculture Co., Ltd. (Shanwei, Guangdong, China). Diseased shrimp showed typical hepatopancreatic atrophy and an empty gastrointestinal tract (GIT). Hemolymph from five shrimp was pooled as one biological sample (*n* = 6), collected from the ventral sinus into pre-cooled anticoagulant, and separated for DNA, RNA, and protein analyses. Hemocytes and hemolymph-associated bacterial pellets were collected by differential centrifugation for nucleic acid extraction, whereas hemocyte pellets or plasma were used for protein and metabolite analyses. A separate batch of healthy shrimp (5–8 g) from Huaxun Aquatic Product Corporation (Shantou, Guangdong, China) was acclimated in aerated artificial seawater (salinity 10‰, 23–26°C) for 2–3 days, fed a commercial diet twice daily, and fasted for 24 h before experiments. All animal experiments were approved by the Animal Research and Ethics Committee of Shantou University and conducted in accordance with institutional guidelines. No human participants were involved.

*Vibrio parahaemolyticus* (MCCC1H00057; GenBank FJ161313.1) and *Shewanella basaltis* (MCCC 1A00286; GenBank NR_044418.1) were obtained from the Marine Culture Collection of China and cultured at 30°C in Tryptic Soy Broth (TSB) and 2216E medium, respectively. *Staphylococcus aureus* (Sa), previously isolated from penaeid shrimp in our laboratory, was cultured in Luria–Bertani (LB) medium.

### Hemocyanin knockdown and bacterial challenge

Double-stranded RNAs (dsRNA) targeting *L. vannamei* hemocyanin (ds*Lv*HMC; GenBank XM_027383261.1) or green fluorescent protein (dsGFP; GenBank U55762.1) were synthesized using T7 promoter-linked primers and the HiScribe T7 Quick High Yield RNA Synthesis Kit (NEB, USA). Shrimp were injected intramuscularly with ds*Lv*HMC or dsGFP (10 μg per shrimp) every 24 h. At 48 h after the first dsRNA injection, shrimp received *V. parahaemolyticus* (2 × 10^5^ colony-forming units (CFU)/shrimp) or sterile 0.65% saline. Knockdown efficiency was assessed by quantitative real-time polymerase chain reaction (qPCR) and Western blot at 24 h post-challenge. Hemolymph was collected from 30 shrimp per group and divided for DNA, RNA, and protein analyses. For metabolomics, plasma from 30 shrimp per group was collected into an equal volume of pre-cooled 0.1 M ethylenediaminetetraacetic acid (EDTA) (pH 7.2) and clarified by centrifugation to remove hemocytes and bacteria.

### RNA and DNA extraction and qPCR

Total RNA from hemocytes and genomic DNA from hemolymph-associated bacteria were extracted using TRIzol Plus RNA Purification Kit (Invitrogen, USA) and TIANamp Marine Animals DNA Kit (TIANGEN, China), respectively. Sample quantity and integrity were assessed using a NanoDrop 2000 spectrophotometer (Thermo, USA), Agilent 2100 Bioanalyzer (Agilent, USA), and agarose gel electrophoresis. First-strand cDNA was synthesized using EasyScript One-Step gDNA Removal and cDNA Synthesis SuperMix (TransGen Biotech, China).

Relative qPCR was performed with gene-specific primers (Table S1), using elongation factor 1 alpha (*Lv*EF-1α) as the internal control and the 2^−ΔΔCT^ method. Absolute qPCR for bacterial quantification used gDNA templates and strain-specific standard curves. Primers targeted total bacteria, *Vibrio* spp., *Shewanella* spp., and *S. aureus* rpoB (Table S1). Reactions were performed with 2× RealStar SYBR Green Mix (Genstar, China) on a qTOWER^3^G system (Analytik Jena, Germany). PCR-based identification of *Vibrio* spp. used *tlh, PirB*, and *Vhh* primers (Table S1). Each assay included three technical replicates.

### Protein extraction and Western blotting

Hemolymph from hemocyanin-knockdown and control shrimp, with or without *V. parahaemolyticus* challenge, was separated into plasma and hemocyte fractions. Hemocytes were lysed in cold NP-40 lysis buffer and clarified by centrifugation. Plasma and lysates were resolved by 10% sodium dodecyl sulfate-polyacrylamide gel electrophoresis (SDS-PAGE), transferred to PVDF membranes (Millipore, USA), blocked with 5% skim milk in TBST, and incubated with rabbit anti-shrimp hemocyanin antibody or mouse anti-α-tubulin antibody (Sigma-Aldrich, USA), followed by horseradish peroxidase (HRP)-conjugated secondary antibodies (Thermo, USA). Signals were detected using enhanced chemiluminescence (ECL) on an Amersham Imager 600 (GE Healthcare, USA). Band intensities were quantified using ImageJ 1.46r (NIH, USA) and normalized to α-tubulin.

### Untargeted metabolomics and data processing

Plasma metabolites were extracted from 100 μl plasma using acetonitrile/methanol (1:1, v/v) containing L-2-chlorophenylalanine as an internal standard. After sonication, protein precipitation, centrifugation, drying, and reconstitution, supernatants were analyzed by liquid chromatography-tandem mass spectrometry (LC–MS/MS). Pooled quality control (QC) samples were prepared by mixing equal volumes of all extracts and injected every 10 runs.

LC–MS/MS was performed on a Thermo UHPLC-Q Exactive HF-X system equipped with an ACQUITY HSS T3 column (100 × 2.1 mm, 1.8 μm). Data-dependent acquisition was conducted in both positive and negative electrospray ionization modes over an *m/z* range of 70–1050. Raw data were processed using Progenesis QI (Waters, USA), with internal standard peaks and known false-positive features removed. Metabolites were annotated against Human Metabolome Database (HMDB), Metlin, and Majorbio databases. The resulting matrix was processed on the Majorbio Cloud platform by retaining features present in at least 80% of samples, imputing low-abundance values with the minimum value, applying sum normalization, excluding QC variables with relative standard deviation (RSD) >30%, and log10-transforming intensities. Partial least squares discriminant analysis (PLS-DA) and seven-cycle cross-validation were performed using the R package ropls (v1.6.2). Differential metabolites were defined as variable importance in projection (VIP) >1 and *P <*.05 by Student’s *t-*test. Raw metabolomics data are available in MetaboLights under accession MTBLS8272.

### Targeted metabolite and enzyme assays

Niacinamide levels in shrimp plasma were measured using an enzyme-linked immunosorbent assay (ELISA) kit (Boshen Biotechnology, China), and NAD levels were quantified using a Coenzyme I NAD(H) Assay Kit (Solarbio, China), following the manufacturers’ protocols. Absorbance was measured with a BioTek Synergy H1 microplate reader (USA). All assays were performed with technical triplicates and three independent biological replicates.

Hemocyanin was purified from shrimp plasma by gel filtration chromatography on an AKTA pure system (GE, USA), and hemocyanin-containing fractions were verified by SDS–PAGE and Western blot. GST-tagged *Lv*NRK1 (XM_027351750.1), *Lv*SIRT2 (XM_027358632.1), and *Lv*SIRT6 (XM_027372938) were cloned into pGEX-6p-1 using the ClonExpress II One Step Cloning Kit (Vazyme, China), expressed as fusion proteins, and incubated with purified hemocyanin at 4°C for GST pull-down analysis. Bound proteins were washed, resolved by SDS–PAGE, and analyzed by Western blotting; 5% input protein was used as a loading control.

NRK1 activity after hemocyanin interaction was estimated from the conversion of nicotinamide riboside (NR) to nicotinamide mononucleotide (NMN). Reaction mixtures containing Tris–HCl, MgCl_2_, ATP, nicotinamide riboside and purified NRK1, hemocyanin, or NRK1–hemocyanin complexes were incubated at 30°C for 30 min, terminated by boiling, and analyzed using an NMN ELISA kit (Boshen Biotechnology, China). Relative NRK1 activity was calculated as the molar conversion rate of nicotinamide riboside to NMN. SIRT2 and SIRT6 activities after hemocyanin interaction were measured using the Epigenase Universal SIRT Activity/Inhibition Assay Kit (EpigenTek, USA) with trichostatin A to suppress histone deacetylase (HDAC) I/II interference. Fluorescence was measured at 530/590 nm (excitation/emission), and activity was calculated from the standard curve as ng/min/mg protein.

### 16S rRNA gene sequencing and microbial community analysis

Hemolymph bacterial DNA was sent to Majorbio Bio-Pharm Technology Co., Ltd. (Shanghai, China) for 16S ribosomal ribonucleic acid (16S rRNA) gene sequencing. The V3–V4 region was amplified using primers 338F/806R (Table S1), purified, pooled in equimolar amounts, and sequenced on an Illumina PE300/250 platform. Raw reads were deposited in the NCBI Sequence Read Archive (SRA) under BioProject PRJNA988426.

Raw reads were demultiplexed, quality-filtered using fastp (v0.19.6), and merged using FLASH (v1.2.7). Operational taxonomic units (OTUs) were clustered at 97% similarity using UPARSE (v7.1), and representative sequences were taxonomically assigned using the RDP Classifier (v2.2) against the SILVA v138 database with a confidence threshold of 0.7. Samples were rarefied to 20 000 reads before diversity analysis. Rarefaction curves, observed OTUs, Chao1 richness, Shannon index, and Good’s coverage were calculated with Mothur (v1.30.1). Principal coordinate analysis (PCoA) based on Bray–Curtis dissimilarity was performed using the R package vegan (v2.5-3) on the Majorbio Cloud platform.

### Niacinamide supplementation and survival assays

For niacinamide supplementation, shrimp were injected with ds*Lv*HMC or dsGFP as described above. At 48 h after knockdown, shrimp received 100 μl niacinamide (0.33 mM) or vehicle control, followed 24 h later by *V. parahaemolyticus, S. aureus* (2 × 10^5^ CFU per shrimp), or sterile saline. Hemolymph from five shrimp per group was collected for bacterial 16S rRNA gene sequencing and qPCR analysis. Survival was monitored for 96 h after niacinamide injection or 48 h after bacterial challenge.

### Statistical analysis

Data are presented as mean ± standard error of the mean (SEM) unless otherwise indicated. Statistical analyses were performed using SPSS v20.0, GraphPad Prism v8.0.1 and the Majorbio Cloud platform. Differences between two groups were assessed using an unpaired two-tailed Student’s *t-*test when data met assumptions of normality and equal variance. Kaplan–Meier survival curves were compared using the log-rank (Mantel–Cox) test. Pearson correlation, linear regression, and Spearman correlation analyses were applied where appropriate. *P <*.05 was considered statistically significant.

## Results

### Shrimp plasma metabolites profile changes with hemocyanin levels

**Figure 1:**
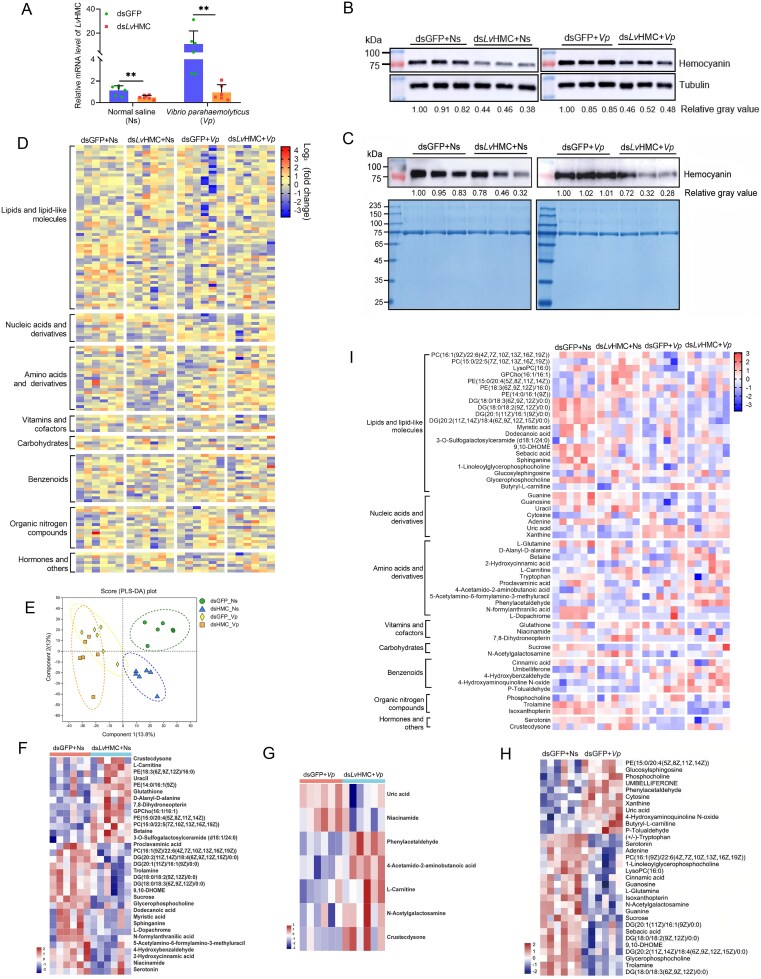
Global metabolic profiles of penaeid shrimp plasma after hemocyanin knockdown under physiological and pathological conditions. (**AB**) Hemocyanin expression in shrimp hemocytes determined by (**A**) qPCR and (**B**) Western blot (WB) analysis after dsRNA-mediated knockdown. (**C**) Hemocyanin protein expression in shrimp plasma after dsRNA-mediated knockdown determined by WB. (**D**) Metabolite categories analyzed by heatmap (*n* = 6). (**E**) Partial least squares discriminant analysis (PLS-DA) based on Bray–Curtis distance. (**F**–**H**) Heatmaps showing significantly dysregulated metabolites in the plasma of (**F**) dsGFP + Ns vs ds*Lv*HMC + Ns shrimp, (**G**) dsGFP + *Vp* vs ds*Lv*HMC + *Vp* shrimp, and (**H**) dsGFP + Ns vs dsGFP + *Vp* shrimp. (**I**) Significantly changed metabolite categories identified from the volcano plots and analyzed by heatmap. The heat map scale represents relative metabolite abundance from low to high levels (*n* = 6).

Given that hemolymph hemocyanin levels affect various metabolic processes in *Penaeus* shrimp [39, 40], we examined plasma metabolite profiles after hemocyanin knockdown with or without *V. parahaemolyticus* challenge (ds*Lv*HMC + *Vp* vs dsGFP + *Vp*; ds*Lv*HMC + Ns vs dsGFP + Ns). Hemocyanin knockdown in hemocytes and plasma was confirmed (Fig. 1A–C). Untargeted metabolomics revealed marked changes in plasma metabolites, with 142 differential metabolites identified across the four groups (Table S2; Fig. 1D). Most belonged to lipids and lipid-like molecules (40.85%), followed by amino acids and derivatives (16.90%), benzenoids (11.97%), and organic nitrogen compounds (10.56%).

PLS-DA showed clear separation among groups (Fig. 1E). Based on OPLS-DA, VIP ≥1, and *t-*test (*P* ≤ .05), 33 differential metabolites were identified in ds*Lv*HMC + Ns versus dsGFP + Ns, 7 in ds*Lv*HMC + *Vp* versus dsGFP + *Vp*, and 32 in dsGFP + *Vp* versus dsGFP + Ns (Fig. 1F–H). These significantly dysregulated metabolites were grouped into eight categories, again dominated by lipids and lipid-like molecules, followed by amino acids and derivatives (Fig. 1I).

### Hemocyanin levels affect niacinamide metabolism

**Figure 2:**
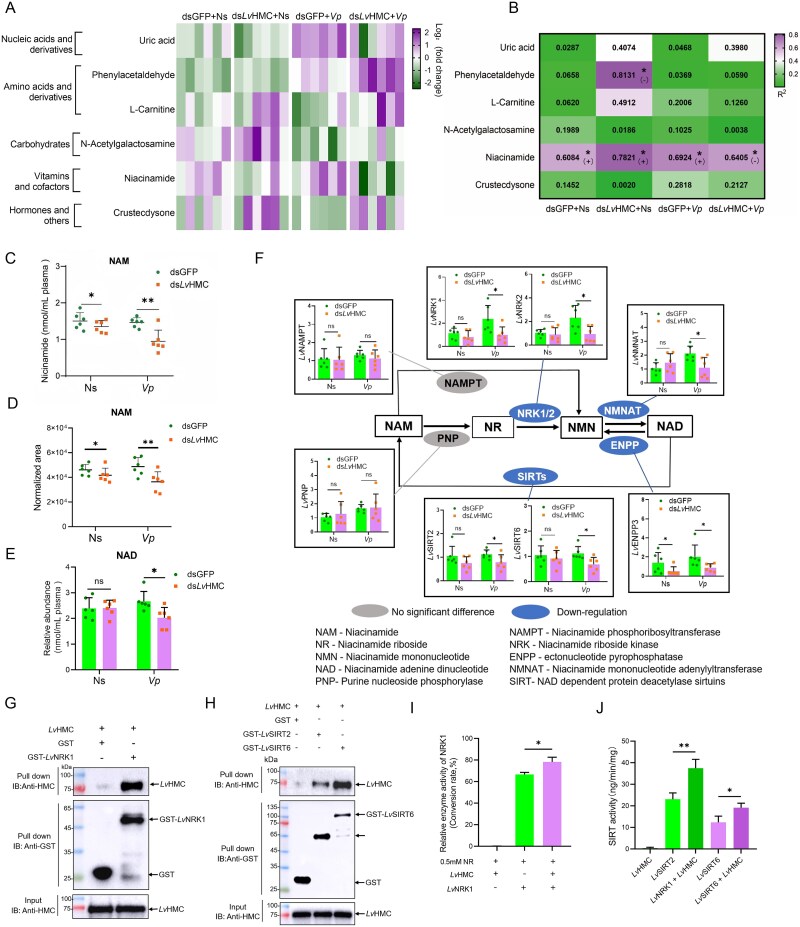
Niacinamide metabolism is dysregulated in shrimp hemolymph after hemocyanin knockdown under pathological conditions. (**A**) Hemocyanin-related metabolites were analyzed by heatmap (*n* = 6). (**B**) Heatmap showing the correlation between hemocyte hemocyanin levels and hemocyanin-related metabolites abundance in shrimp plasma determined. Statistical significance is indicated by asterisks (^*^P < .05, ^**^P < .01, ns means not significant). The plus sign represents positive correlation and the minus sign represents negative correlation. (**C**) Shrimp plasma niacinamide level determined by ELISA. (**D**) Violin diagram showing the differential expression of niacinamide in shrimp plasma determined by GC–MS analysis. (**E**) Shrimp plasma NAD level determined by ELISA. (**F**) Schematic diagram showing changes in niacinamide salvage pathway–related enzymes at the transcriptional level in shrimp hemocytes after hemocyanin knockdown under physiological and pathological conditions. (**GH**) Immunoblots of protein–protein interaction analysis between (**G**) *Lv*HMC and GST-*Lv*NRK1 or (**H**) *Lv*HMC, GST-*Lv*SIRT2, and GST-*Lv*SIRT6. (**I**) Relative enzymatic activity of *Lv*NRK1 [expressed as the conversion rate of nicotinamide riboside (NR) to nicotinamide mononucleotide (NMN)] under 0.5 mM NR substrate condition following interaction with *Lv*HMC. (**J**) Enzymatic activity of *Lv*SIRT2 and *Lv*SIRT6 following interaction with *Lv*HMC. Each dot represents an independent sample (*n* = 6), and results were reported as mean ± SEM. ^*^P < .05, ^**^P < .01.

To identify metabolites consistently associated with hemocyanin under physiological and pathological conditions, we compared ds*Lv*HMC + Ns and ds*Lv*HMC + *Vp* with their respective controls and obtained six shared differential metabolites (Fig. 2A). Among them, niacinamide showed the strongest association with hemocyanin expression. Hemocyte hemocyanin levels were positively correlated with plasma niacinamide in dsGFP + Ns, ds*Lv*HMC + Ns, and dsGFP + *Vp*, but negatively correlated in ds*Lv*HMC + *Vp* (Fig. 2B). ELISA and LC–MS/MS further confirmed that hemocyanin knockdown significantly reduced niacinamide levels, particularly after pathogen challenge (Fig. 2C and D).

We next examined key enzymes in the niacinamide salvage pathway. Under *V. parahaemolyticus* challenge, hemocyanin knockdown significantly reduced the hemocyte transcript levels of NRK1/2, NMNAT, ENPP1, SIRT2, and SIRT6, and also decreased plasma NAD levels (Fig. 2E and F). Pull-down assays showed that *Lv*HMC interacted with *Lv*NRK1, *Lv*SIRT2, and *Lv*SIRT6 (Fig. 2G and H). These interactions significantly enhanced *Lv*NRK1 catalytic conversion of NR to NMN, as well as *Lv*SIRT2 and *Lv*SIRT6 enzymatic activities (Fig. 2I and J). Together, these results indicate that hemocyanin regulates niacinamide metabolism during pathogen challenge.

### Hemolymph microbiota composition is modulated by hemocyanin levels

**Figure 3:**
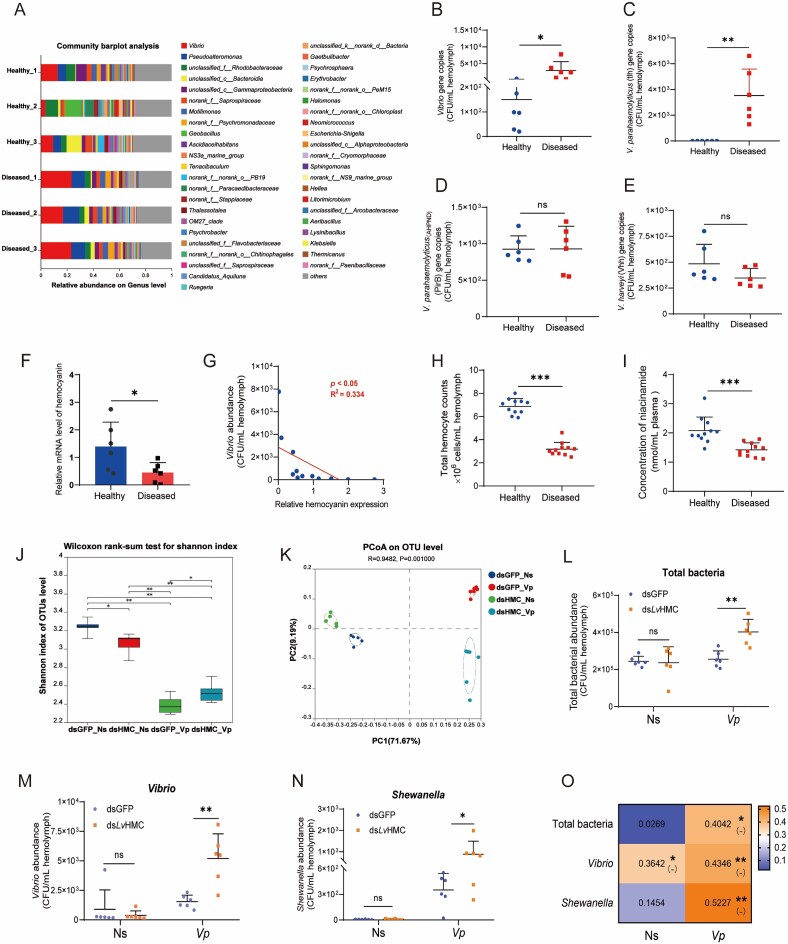
Hemocyanin level affects microbial composition and *Vibrio* abundance in the hemolymph of penaeid shrimp. (**A**) Community bar plot of proportions of bacteria taxa in the hemolymph of healthy and diseased penaeid shrimp at genus level (*n* = 3). (**B**–**F**) PCR-based quantification of the 16S rRNA gene in shrimp hemolymph including (**B**) *Vibrio* abundance, (**C**) *V. parahaemolyticus* (*tlh*), (**D**) *V. parahaemolyticus*  _(AHPND)_ (*pirB*), and (**E**) *V. harveyi* (*vhh*). (**F**) Hemocyanin transcript levels in hemocytes. (**G**) Correlation between hemocytes hemocyanin transcript levels and *Vibrio* abundance in the hemolymph of healthy and diseased shrimp. (**H**) Total hemocyte counts in the hemolymph of healthy and diseased shrimp. (**I**) Relative abundance of niacinamide in shrimp plasma determined by ELISA. (**JK**) Hemolymph microbial community diversity at the OTU level after hemocyanin knockdown under physiological conditions and pathogen challenge conditions determined using (**J**) Shannon diversity index and (**K**) principal coordinates analysis (PCoA). (**L**–**N**) The quantification of the (**L**) total bacterial abundance, (**M**) *Vibrio* abundance, (**N**) *Shewanella* abundance after dsRNA (dsGFP or ds*Lv*HMC) injection by qPCR. (**O**) Correlation between hemocyte hemocyanin levels and the abundance of total bacterial abundance, *Vibrio* abundance, and *Shewanella* analyzed by heatmap. Dots represent independent biological samples. Results were reported as mean ± SEM (*n* = 6). Statistical significance is indicated by asterisks (^*^P < .05, ^**^P < .01, ns means not significant).

Given the effect of hemocyanin on niacinamide metabolism, we next analyzed hemolymph microbiota by 16S rRNA sequencing in healthy and diseased shrimp collected from the same pond. At the genus level, diseased shrimp showed a markedly higher relative abundance of *Vibrio* than healthy shrimp (21.27% vs 8.71%; Fig. 3A), and qPCR confirmed significantly higher absolute *Vibrio* abundance in diseased shrimp (Fig. 3B). Among the pathogen-specific markers tested, only the *tlh* gene of *V. parahaemolyticus* was significantly elevated in diseased shrimp, whereas *pirB* and *vhh* did not differ significantly (Fig. 3C–E). Diseased shrimp also exhibited lower hemocyte hemocyanin transcript levels, which were negatively correlated with hemolymph *Vibrio* abundance (Fig. 3F and G). In addition, diseased shrimp had lower total hemocyte counts and lower plasma niacinamide levels than healthy shrimp (Fig. 3H and I).

We then examined the effect of hemocyanin knockdown on hemolymph microbiota with or without pathogen challenge. Shannon diversity decreased significantly after *V. parahaemolyticus* challenge in both control and hemocyanin-silenced shrimp, and also declined in ds*Lv*HMC + Ns relative to dsGFP + Ns (Fig. 3J). PCoA showed distinct bacterial communities among the four groups (Fig. 3K). Under pathogen challenge, hemocyanin knockdown significantly increased total bacterial abundance as well as the relative abundances of *Vibrio* and *Shewanella* (Fig. 3L–N). Hemocyte hemocyanin transcript levels were negatively correlated with total bacteria, *Vibrio*, and *Shewanella* abundance (Fig. 3O), indicating that reduced hemocyanin favors the expansion of opportunistic pathogens during *V. parahaemolyticus* infection.

Correlation analysis between dysregulated metabolites and altered bacterial taxa further showed that, after hemocyanin knockdown and *V. parahaemolyticus* stimulation, niacinamide was negatively correlated with *Vibrio, Rheinheimera, Shewanella*, and *Flavobacterium*, with the strongest association observed for *Vibrio* (Fig. S1).

### Hemolymph microbiota composition is modulated by hemocyanin via niacinamide metabolism during *Vibrio* challenge

**Figure 4:**
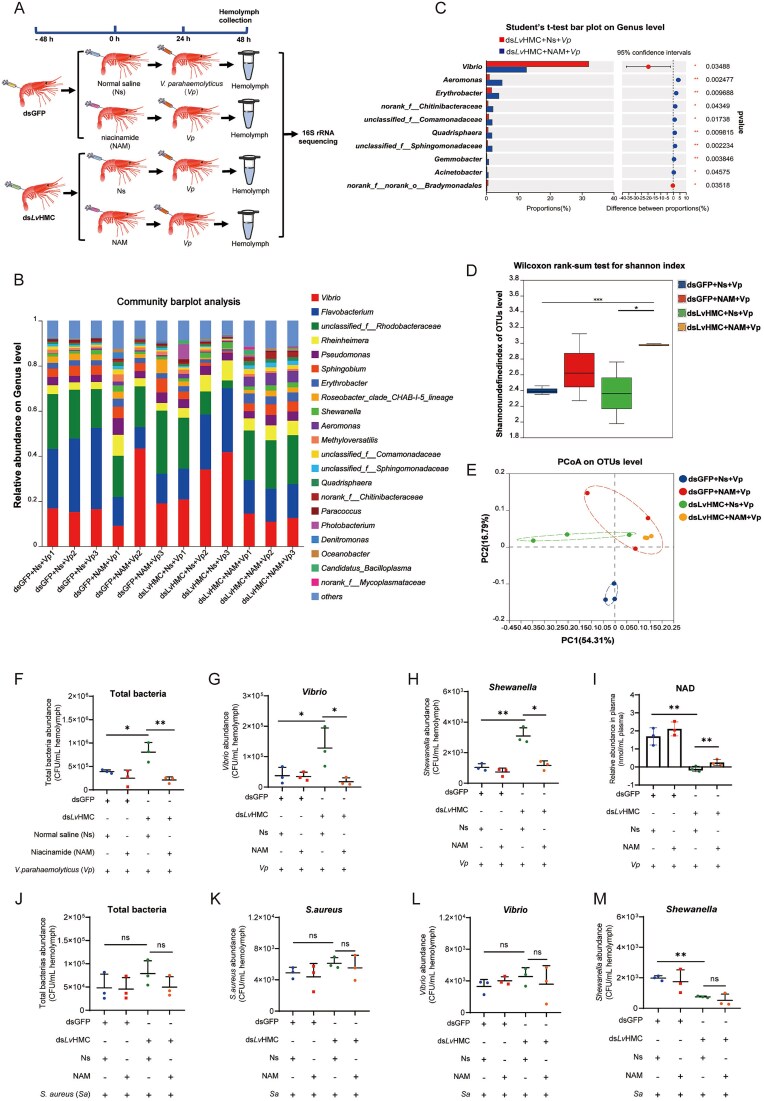
Niacinamide supplementation shapes the hemolymph microbiota in *Vibrio*-infected shrimp. (**A**) Exogenous treatment of *L. vannamei* with or without niacinamide (NAM) after depleting hemocyanin followed by *V. parahaemolyticus* (*Vp*) infection. (**BC**) Relative bacteria abundance in hemolymph at the genus level after hemocyanin knockdown (48 hpi) with and without NAM treatment (24 hpi) followed by *Vp* infection (24 hpi) shown by (**B**) community bar plot of bacteria taxa proportions and (**C**) Student’s *t*-test bar plot at genus level (*n* = 3). (**DE**) Hemolymph microbial community diversity at the OTU level after hemocyanin knockdown with or without NAM supplementation followed by *V. parahaemolyticus* (*Vp*) infection using (**D**) Shannon diversity index and (**E**) principal coordinates analysis (PCoA). (**F**–**H**) the quantification of the (**F**) total bacterial abundance, (**G**) *Vibrio* abundance, and (**H**) *Shewanella* abundance using qPCR. (**I**) The NAD levels in shrimp plasma were determined by ELISA. (**J**–**M**) the quantification of the (**J**) total bacterial abundance, (**K**) *Staphylococcus aureus*, (**L**) *Vibrio* abundance, and (**M**) *Shewanella* abundance using qPCR after hemocyanin knockdown (48 hpi) with and without NAM treatment (24 hpi) followed by *S. aureus* (*Sa*) infection (24 hpi). Each dot represents an independent sample (*n* = 3), and results were reported as mean ± SEM. ^*^P < .05, ^**^P < .01.

To test whether hemocyanin regulates hemolymph microbiota through niacinamide, hemocyanin-silenced shrimp were supplemented with niacinamide and then challenged with *V. parahaemolyticus* (Fig. 4A). 16S rDNA sequencing showed distinct bacterial communities among the four treatment groups (Fig. 4B). In hemocyanin-silenced shrimp without niacinamide supplementation, *Vibrio* became the dominant genus (32.14%), whereas niacinamide supplementation reduced its proportion to 12.64% (Fig. 4C). Niacinamide also significantly increased bacterial alpha diversity in hemocyanin-silenced shrimp after *V. parahaemolyticus* challenge (Fig. 4D), and PCoA revealed clear group separation (Fig. 4E). Consistently, qPCR showed that niacinamide supplementation reduced total bacterial abundance and the relative abundances of *Vibrio* and *Shewanella* in hemocyanin-silenced shrimp (Fig. 4F–H). The reduced plasma NAD level caused by hemocyanin knockdown was also restored by niacinamide supplementation (Fig. 4I).

We further asked whether this regulation also occurred during Gram-positive bacterial infection. After *Staphylococcus aureus* challenge, niacinamide supplementation did not significantly alter the abundances of total bacteria, *S. aureus*, or *Vibrio*, although *Shewanella* abundance was lower in ds*Lv*HMC + Ns + *Sa* than in dsGFP + Ns + *Sa* (Fig. 4J–M). These results suggest that hemocyanin-dependent niacinamide regulation of hemolymph microbiota is more evident during *V. parahaemolyticus* infection than during *S. aureus* infection.

### Niacinamide enhances shrimp survival during *Vibrio parahaemolyticus* infection

**Figure 5:**
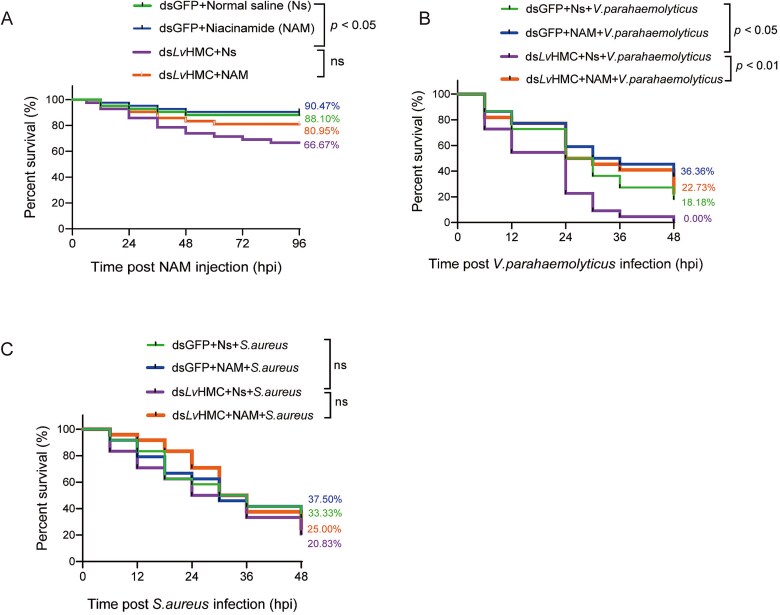
Effect of niacinamide (NAM) supplementation on penaeid shrimp survival. Shrimp survival rate (**A**) after hemocyanin knockdown with and without NAM treatment under physiological conditions, and (**B**) after hemocyanin knockdown with and without NAM treatment followed by *V. parahaemolyticus* (*Vp*) or (**C**) *S. aureus* (*Sa*) infection was recorded. The product-limit method of Kaplan–Meier was used to calculate the shrimp survival rate and the significance compared using the log-rank test.

Finally, we assessed whether niacinamide affects shrimp survival after hemocyanin knockdown. Under physiological conditions, niacinamide modestly improved survival of hemocyanin-silenced shrimp (80.95% vs 66.67%; Fig. 5A). After *V. parahaemolyticus* infection, survival in the ds*Lv*HMC + Ns group fell to 0% within 48 h, whereas niacinamide supplementation increased survival to 22.73% (Fig. 5B). In contrast, after *S. aureus* infection, niacinamide had only a limited effect on survival (25.00% vs 20.83%; Fig. 5C). These findings indicate that hemocyanin-mediated niacinamide regulation contributes to shrimp resistance to *V. parahaemolyticus* infection.

## Discussion

Crustaceans possess a semi-open circulatory system in which hemolymph is continuously exposed to microorganisms from the surrounding aquatic environment [41, 42]. Under normal conditions, hemolymph microbiota are maintained in a relatively stable state, whereas environmental stress or pathogen invasion can disrupt this balance and promote the expansion of opportunistic bacteria [15, 42]. In the present study, we show that this homeostasis is closely linked to hemocyanin, the most abundant soluble protein in shrimp hemolymph. Diseased shrimp exhibited lower hemocyte hemocyanin expression, reduced niacinamide levels, and increased *Vibrio* abundance, while experimental hemocyanin knockdown reproduced these changes and further promoted the proliferation of *Vibrio* and *Shewanella* during *V. parahaemolyticus* challenge. These findings support a role for hemocyanin in maintaining hemolymph microbial balance under infection pressure.

Hemocyanin is best known as a respiratory protein, but accumulating evidence indicates that it also participates broadly in shrimp immune regulation [43–45]. Given that some metabolites play an essential role in maintaining microbiome homeostasis and impact host susceptibility to pathogenic microorganisms [5, 46] or enhance host immune surveillance [47], it is plausible that shrimp hemocyanin could affect metabolic processes in hemolymph to affect the microbiota since hemocyanin has previously been implicated in fatty acid metabolism [40] and energy metabolism [39] in the hepatopancreas. Thus, the current study explored the changes in metabolite profiles and the consequences on hemolymph microbiota composition in diseased and healthy penaeid shrimp, which expressed different levels of hemocyanin.

After metabolomic profiling of hemolymph samples from *V. parahaemolyticus*–infected and healthy penaeid shrimp, which expressed different levels of hemocyanin, six dysregulated metabolites (uric acid, phenylacetaldehyde, L-carnitine, *N*-acetylgalactosamine, niacinamide, and crustecdysone) that seem to play a crucial role in shrimp susceptibility to *Vibrio* were identified. Indeed, strong positive correlations were observed between the relative levels of hemocyanin and two key metabolites, i.e. uric acid and niacinamide, with the latter being the strongest (Fig. 2B). Niacinamide, which is an amide derivative of water-soluble vitamin B_3_, is regarded as a safe therapeutic agent for the treatment of various human diseases, including melanoma [48], diabetes mellitus [49], and Parkinson’s disease [50]. As a major precursor in the biosynthesis of NAD in mammalian cells [24], the conversion of niacinamide into niacinamide mononucleotide (NMN) through the salvage pathway constitutes the primary route by which NAD is produced [51]. On the other hand, NAD is a crucial substrate for essential enzymes, such as SIRT, PARP, and CD38 [25], which is used in metabolism and niacinamide production. In the present study, mRNA transcript levels of rate-limiting enzymes of the niacinamide salvage pathway, i.e. NRK1/2, NMNAT, ENPP1, and SIRT2/6, and plasma NAD levels were attenuated as a result of depleted hemocyanin (*Lv*HMC) expression during *V. parahaemolyticus* infection. Moreover, *Lv*HMC was found to interact with *Lv*NRK1 (Fig. 2G), *Lv*SIRT2, and *Lv*SIRT6 (Fig. 2F-H), and this physical interaction was accompanied by a significant enhancement of the catalytic activities of these niacinamide salvage pathway enzymes (e.g. NRK1, SIRT2, SIRT6) (Fig. 2I and J), suggesting that hemocyanin modulates the niacinamide metabolism and hemocyanin depletion results in the niacinamide salvage pathway metabolism perturbation.

We found that penaeid shrimp (*L. vannamei*) in the same aquaculture pond that displayed characteristic symptoms (atrophied hepatopancreases with loose hepatic tubules and hemocytic infiltration, shedding of hepatic tubules, and empty GITs) of *Vibrio* infection [52, 53] had low hemocyte hemocyanin levels, coupled with negative correlation with *Vibrio* abundance (Fig. 3A–G). Interestingly, significantly lower levels of niacinamide were also found in the plasma of diseased shrimp compared with healthy shrimp (Fig. 3I), suggesting that niacinamide levels are modulated by hemocyanin to enhance shrimp resistance to *Vibrio* infection. Although there is currently limited information on the role of niacinamide in microbiota regulation, niacinamide has activity against a variety of pathogenic microorganisms, including *Plasmodium falciparum* [54], *Mycobacterium tuberculosis* [55], and *Candida albicans* [56]. Incubation of niacinamide with African *Trypanosomiasis* resulted in lysosome destruction, cytoplasmic division, and cell death [57]. Our data indicate that niacinamide supplementation significantly attenuates *Vibrio* levels caused by decreased hemocyanin expression under *V. parahaemolyticus* infection. Interestingly, the effects of hemocyanin on hemolymph homeostasis through niacinamide metabolism was induced by *V. parahaemolyticus* (Gram-negative bacteria), while the response to *S. aureus* (Gram-positive bacteria) was not significant (Fig. 4), suggesting that niacinamide may function as an antimicrobial metabolite against Gram-negative bacteria. In the hemolymph of most aquatic invertebrates, such as the Pacific oyster *Crassostrea gigas* [58], mud crab *Scylla paramamosian* [59], and scallop *Argopecten purpuratus* [13], the abundant and dominant bacterial genera are mainly Gram negative, with *Vibrio* being the most common opportunistic pathogen [60]. Thus, hemocyanin, shrimp’s most abundant plasma protein, may represent an innate immune strategy against Gram-negative bacteria exerted via niacinamide metabolism in crustaceans.

**Figure 6:**
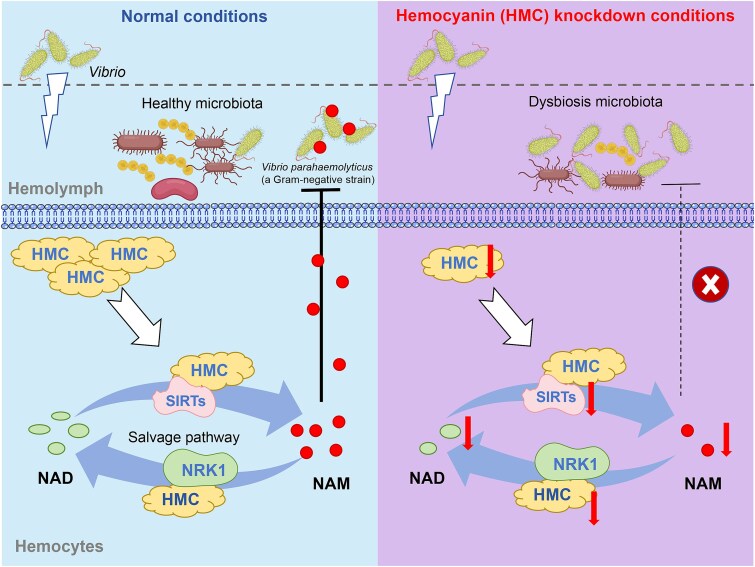
Proposed schematic illustration of the mechanism by which hemocyanin-modulated niacinamide shapes the hemolymph microbiota in *Vibrio*-infected shrimp. Under normal conditions, *Lv*HMC maintains the homeostasis of the nicotinamide salvage pathway. Knockdown of *Lv*HMC level causes metabolic disorder of the nicotinamide salvage pathway, which further impairs the host immune defense and ultimately increases the susceptibility of *L. vannamei* to *V. parahaemolyticus* infection.

In conclusion, we revealed that hemocyanin modulates microbial composition in the hemolymph of penaeid shrimp during *V. parahaemolyticus* infection. The proposed mechanism supporting the current findings is that decreased hemocyanin levels induce the niacinamide salvage pathway metabolism perturbation, which consequently causes *V. parahaemolyticus* infection in penaeid shrimp (Fig. 6).

## Supplementary Material

ycag119_Supplementary_Figure_and_Tables

## Data Availability

The 16S rRNA gene sequencing data generated during this study are available at the NCBI Sequence Read Archive with BioProject accession numbers PRJNA988426, while all the metabolomic raw data are deposited to MetaboLights (http://www.ebi.ac.uk/metabolights/) with the unique identifier MTBLS8272.
