# Supplementary material for: Niacinamide modulation by hemocyanin shapes hemolymph microbial communities in Penaeid shrimp
Source: ISME Commun. 2026 May 7;6(1):ycag119. doi: 10.1093/ismeco/ycag119 (PMC13245178; doi:10.1093/ismeco/ycag119)
Supplement: ycag119_Supplementary_Figure_and_Tables [file ycag119_supplementary_figure_and_tables.pdf]

Supplementary Figure

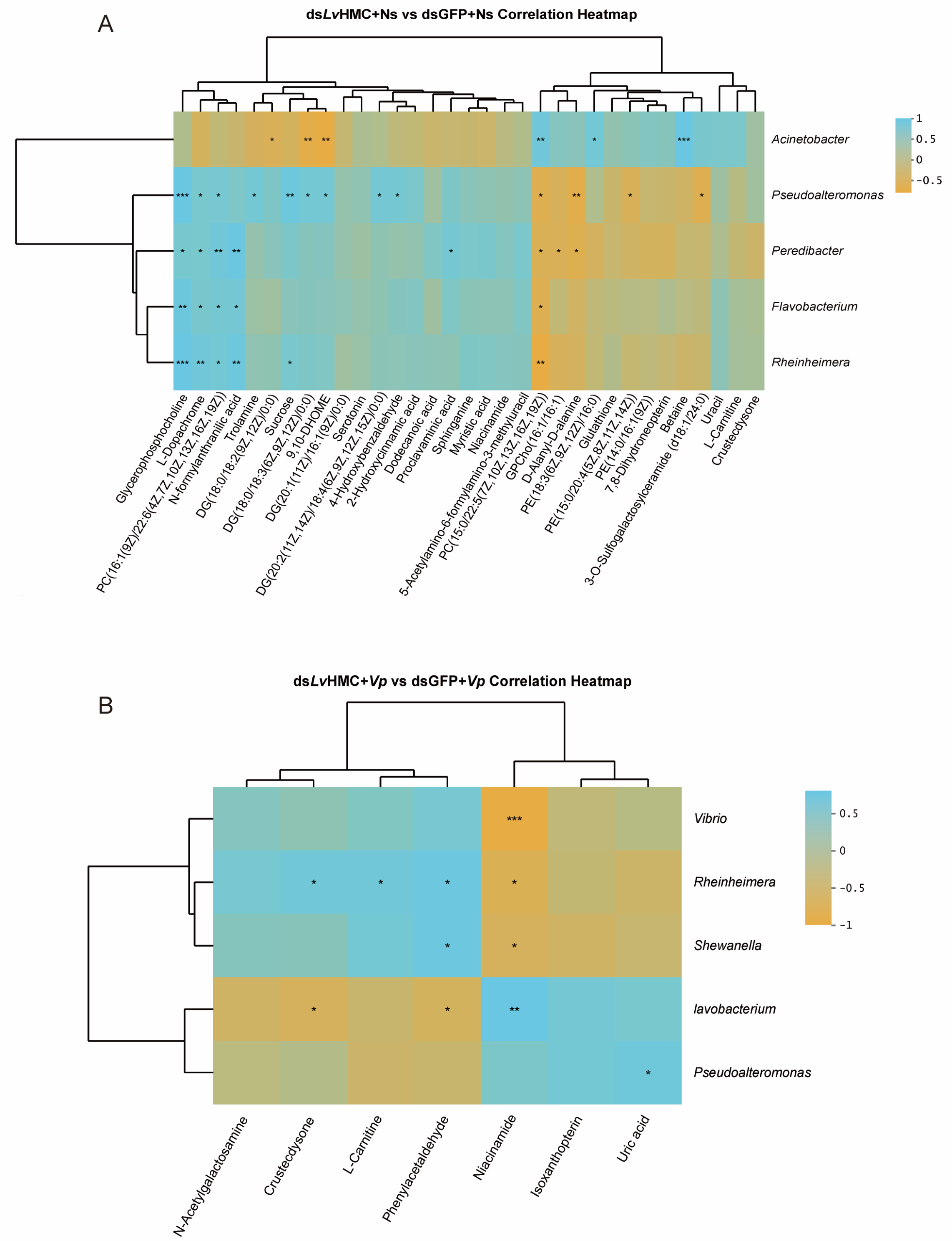

Figure S1. Spearman correlation analysis between significantly dysregulated metabolites in the plasma and significantly changed bacteria species (at genus level) in the hemolymph of **(A)** ds*Lv*HMC+Ns vs dsGFP+Ns shrimp and **(B)** ds*Lv*HMC+*Vp* vs dsGFP+*Vp* shrimp. Statistical significance is indicated by asterisks (**p* < 0.05, ***p* < 0.01, ****p* < 0.001).

Supplementary Table 1. Normalized area of metabolites in *L. vannamei* plasma

No.	Category of metabolites	Metabolite name	dsGFP+Ns	ds*Lv*HMC+Ns	dsGFP+*Vp*	ds*Lv*HMC+*Vp*
1	Lipids and lipid-like molecules	PC(16:0/18:1(11Z))	175143441.13	179327738.75	193450694.77	160395916.31
2	Lipids and lipid-like molecules	9,10-DHOME	31416246.74	29535870.85	29418424.71	29114228.68
3	Lipids and lipid-like molecules	C16 Sphinganine	26485148.50	15991406.24	18238588.82	15596669.64
4	Lipids and lipid-like molecules	Sebacic acid	19969996.79	19056939.98	19130853.28	19101464.27
5	Lipids and lipid-like molecules	PC(16:0/18:2(9Z,12Z))	19087641.08	18549487.33	19110280.37	17391173.57
6	Lipids and lipid-like molecules	PC(18:0/20:5(5Z,8Z,11Z,14Z,17Z))	18957171.93	22199370.09	20038528.49	17831689.49
7	Lipids and lipid-like molecules	DG(18:0/18:3(6Z,9Z,12Z)/0:0)	18718135.35	17315492.11	17209592.85	16825721.39
8	Lipids and lipid-like molecules	DG(18:0/18:2(9Z,12Z)/0:0)	17080529.96	16273716.90	16231333.98	15893045.00
9	Lipids and lipid-like molecules	PC(16:1(9Z)/P-18:1(11Z))	15968396.45	17197648.67	18907759.01	16034549.19
10	Lipids and lipid-like molecules	PC(18:0/20:4(8Z,11Z,14Z,17Z))	13798672.88	17367624.51	16253346.82	13384290.73
11	Lipids and lipid-like molecules	PS(16:0/20:0)	13065855.20	13501311.06	14569203.41	12202199.01
12	Lipids and lipid-like molecules	Floionolic acid	11098546.14	10844642.44	10967213.89	11214603.17
13	Lipids and lipid-like molecules	PC(16:1(9Z)/22:6(4Z,7Z,10Z,13Z,16Z,19Z))	11144102.90	8531833.42	8563314.38	7265360.28
14	Lipids and lipid-like molecules	PC(16:0/18:3(6Z,9Z,12Z))	8081128.36	8254788.37	8333856.65	7577944.53
15	Lipids and lipid-like molecules	Sphinganine	7504803.83	4736792.29	4907971.36	3963569.83

16	Lipids and lipid-like molecules	(13E)-11a-Hydroxy-9,15-dioxoprost-13-enoic acid	6523771.45	6370480.43	6503510.60	6662431.75
17	Lipids and lipid-like molecules	PC(18:1(11Z)/20:5(5Z,8Z,11Z,14Z,17Z))	4227145.73	4188381.35	3300211.90	3502708.99
18	Lipids and lipid-like molecules	PC(16:0/20:5(5Z,8Z,11Z,14Z,17Z))	4178004.05	4244830.32	3465193.40	3703692.30
19	Lipids and lipid-like molecules	PC(15:0/22:5(7Z,10Z,13Z,16Z,19Z))	3168870.72	4569750.50	4031584.00	3754594.41
20	Lipids and lipid-like molecules	PC(18:2(9Z,12Z)/22:6(4Z,7Z,10Z,13Z,16Z,19Z))	3039654.87	3888580.10	3200615.11	2553721.25
21	Lipids and lipid-like molecules	PC(14:0/20:3(5Z,8Z,11Z))	2770432.23	2739930.17	2623091.97	2139003.08
22	Lipids and lipid-like molecules	PE(22:5(4Z,7Z,10Z,13Z,16Z)/18:0)	2692540.22	3367305.47	2829331.99	2459740.79
23	Lipids and lipid-like molecules	1-Hexanol	2928091.97	1819402.92	1985548.15	1654892.33
24	Lipids and lipid-like molecules	Myristic acid	2826087.23	1662801.86	1916458.53	1474073.43
25	Lipids and lipid-like molecules	GPCho(16:1/16:1)	2017657.24	2370119.77	2187788.07	2047590.00
26	Lipids and lipid-like molecules	PE(15:0/20:4(5Z,8Z,11Z,14Z))	1983119.10	2484213.50	2273032.69	2182098.54
27	Lipids and lipid-like molecules	1-Linoleoylglycerophosphocholine	2013525.12	1519830.61	1239997.52	1108936.29
28	Lipids and lipid-like molecules	PE(18:3(6Z,9Z,12Z)/16:0)	1577827.18	1619402.98	1592464.92	1614994.48
29	Lipids and lipid-like molecules	DG(20:1(11Z)/16:1(9Z)/0:0)	1431688.64	1301457.66	1285130.92	1272220.82
30	Lipids and lipid-like molecules	PC(18:3(6Z,9Z,12Z)/P-16:0)	1356430.02	1391406.33	1554916.56	1356225.04
31	Lipids and lipid-like molecules	PC(15:0/18:2(9Z,12Z))	1240044.36	1192031.46	1246633.52	1159497.18
32	Lipids and lipid-like molecules	PE-NMe2(16:0/18:1(9Z))	1199804.05	1125328.47	1260069.92	1058096.76

33	Lipids and lipid-like molecules	Prostaglandin D1	1139347.04	1133268.49	1135676.37	1174036.93
34	Lipids and lipid-like molecules	Glucosylsphingosine	1088907.42	1234235.30	1181436.29	1195191.42
35	Lipids and lipid-like molecules	PE(18:0/20:5(5Z,8Z,11Z,14Z,17Z))	1122038.26	832767.39	1050381.92	854084.20
36	Lipids and lipid-like molecules	DG(18:0/18:4(6Z,9Z,12Z,15Z)/0:0)	1048187.90	1015740.31	989412.19	1025193.62
37	Lipids and lipid-like molecules	SM(d18:0/16:1(9Z))	1044150.12	858583.98	1174525.27	750648.27
38	Lipids and lipid-like molecules	LysoPC(16:0)	1003909.43	1121745.48	705158.14	599722.37
39	Lipids and lipid-like molecules	PC(18:1(11Z)/22:6(4Z,7Z,10Z,13Z,16Z,19Z))	901499.42	820679.86	676924.17	693309.26
40	Lipids and lipid-like molecules	DG(20:2(11Z,14Z)/18:4(6Z,9Z,12Z,15Z)/0:0)	852897.33	800528.71	786807.80	771768.76
41	Lipids and lipid-like molecules	PE(O-18:1(1Z)/20:4(5Z,8Z,11Z,14Z))	744635.00	792637.51	885739.47	720670.27
42	Lipids and lipid-like molecules	PE(14:0/16:1(9Z))	589999.12	1120495.07	861694.95	912222.27
43	Lipids and lipid-like molecules	PE(18:1(11Z)/18:2(9Z,12Z))	528143.94	471398.08	432538.50	488675.60
44	Lipids and lipid-like molecules	3-Oxododecanoic acid	512678.26	511615.27	512536.37	522142.33
45	Lipids and lipid-like molecules	Dodecanoic acid	563472.54	379542.17	433191.71	358421.71
46	Lipids and lipid-like molecules	GPEtn(14:0/22:5)	314486.90	497279.98	338141.76	438890.33
47	Lipids and lipid-like molecules	Phytosphingosine	305973.95	314194.26	281682.99	280222.22
48	Lipids and lipid-like molecules	PE(16:0/20:5(5Z,8Z,11Z,14Z,17Z))	284131.01	380071.92	266556.66	367022.60
49	Lipids and lipid-like molecules	PE(18:0/18:2(9Z,12Z))	267786.44	362885.87	273382.63	321930.98

50	Lipids and lipid-like molecules	Glycerophosphocholine	224451.23	150735.37	124956.66	118389.87
51	Lipids and lipid-like molecules	(+)-Camphor	215828.65	217547.20	217005.35	226997.71
52	Lipids and lipid-like molecules	Geranylacetone	163042.67	166330.79	166812.44	173014.74
53	Lipids and lipid-like molecules	Acetylcarnitine	146571.76	153004.90	150915.77	216786.69
54	Lipids and lipid-like molecules	Gamma-Terpinene	144223.99	146436.09	145528.87	154082.99
55	Lipids and lipid-like molecules	Petunidin	128092.90	141838.40	109337.14	113128.27
56	Lipids and lipid-like molecules	3-O-Sulfogalactosylceramide (d18:1/24:0)	102123.05	228468.13	130421.85	210913.76
57	Lipids and lipid-like molecules	Decanoyl-L-carnitine	40669.94	27302.14	40962.09	73674.52
58	Lipids and lipid-like molecules	Butyryl-L-carnitine	23140.33	18326.05	32113.49	88578.67
59	Nucleic acids and derivatives	Hypoxanthine	3306855.08	3094652.61	3436192.56	3435888.50
60	Nucleic acids and derivatives	Inosine	888987.78	848457.61	866216.69	843819.72
61	Nucleic acids and derivatives	Guanine	404297.41	342365.64	279638.47	248966.76
62	Nucleic acids and derivatives	Guanosine	188559.26	164389.62	128772.49	128154.67
63	Nucleic acids and derivatives	Uric acid	182538.37	144288.47	391393.41	360737.47
64	Nucleic acids and derivatives	Uracil	92396.42	104533.67	85599.52	77853.68
65	Nucleic acids and derivatives	Thymine	34100.70	31867.39	30434.78	27466.90
66	Nucleic acids and derivatives	Cytosine	29978.15	34308.45	35321.71	35614.64

67	Nucleic acids and derivatives	Adenine	23076.48	21795.12	21764.53	20733.39
68	Nucleic acids and derivatives	Xanthine	1597.75	1790.08	10895.70	12435.35
69	Amino acids and derivates	Betaine	11353801.07	13052920.46	10825762.77	14930586.99
70	Amino acids and derivates	Phenylpyruvic acid	2054288.68	2049772.01	2076111.42	2165335.09
71	Amino acids and derivates	Indole	1862281.07	1878476.84	1927342.17	2020417.40
72	Amino acids and derivates	4-Hydroxyphenylacetaldehyde	1590341.73	1531521.08	1540396.29	1554973.00
73	Amino acids and derivates	N6-Acetyl-L-lysine	1027741.73	1010649.31	1014989.42	1046896.28
74	Amino acids and derivates	L-Arginine	844819.42	817824.58	883189.04	963523.27
75	Amino acids and derivates	2-Hydroxycinnamic acid	584201.64	433994.18	582129.12	683314.10
76	Amino acids and derivates	L-Proline	547169.03	370010.28	390261.02	412012.08
77	Amino acids and derivates	N6,N6,N6-Trimethyl-L-lysine	488930.84	540400.72	456779.39	453564.51
78	Amino acids and derivates	L-Carnitine	417579.61	478159.61	391123.71	469554.71
79	Amino acids and derivates	6-Hydroxykynurenic acid	206219.11	126970.76	177814.87	192541.84
80	Amino acids and derivates	(+/-)-Tryptophan	177344.09	169469.56	158068.08	127964.35
81	Amino acids and derivates	2-(Formamido)-N1-(5-phospho-D-ribosyl)acetamidine	170504.46	177534.77	166468.25	166652.03
82	Amino acids and derivates	Proclavaminic acid	140522.53	89513.17	129696.57	132213.00
83	Amino acids and derivates	D-Alanyl-D-alanine	98788.94	106624.63	103023.44	107755.21

84	Amino acids and derivates	7-Chloro-L-tryptophan	80860.89	73392.77	82973.69	86632.03
85	Amino acids and derivates	L-Glutamine	64719.90	46073.82	39525.72	35646.06
86	Amino acids and derivates	4-Acetamido-2-aminobutanoic acid	43063.78	51091.46	47165.71	60112.95
87	Amino acids and derivates	Phenylacetaldehyde	20703.92	22315.98	30619.35	44273.01
88	Amino acids and derivates	Hydroxyphenylacetylglycine	101386.75	37013.56	54877.28	26076.80
89	Amino acids and derivates	Xanthurenic acid	10665.95	6056.75	9449.04	10359.39
90	Amino acids and derivates	5-Acetylamino-6-formylamino-3-methyluracil	12911.61	5564.17	16038.79	14442.89
91	Amino acids and derivates	L-Dopachrome	5196.63	446.15	1139.52	15696.49
92	Amino acids and derivates	N-formylanthranilic acid	5608.52	108.19	958.77	11673.26
93	Vitamins and cofactors	Trigonelline	1887817.58	2195418.04	1840683.08	2216917.07
94	Vitamins and cofactors	P-Aminobenzoic acid	1396725.31	1420347.55	1420012.32	1496932.57
95	Vitamins and cofactors	Glutathione	348337.33	504228.87	460596.27	421293.46
96	Vitamins and cofactors	Pantothenic Acid	181100.56	163988.76	165181.74	159643.16
97	Vitamins and cofactors	Niacinamide	46071.49	41725.96	46270.96	42350.26
98	Vitamins and cofactors	7,8-Dihydroneopterin	5770.37	53650.92	12344.46	46114.77
99	Carbohydrates	N-Acetyl-b-glucosaminylamine	584508.78	583305.65	591143.46	602929.13
100	Carbohydrates	2-O-(6-Phospho-alpha-mannosyl)-D-glycerate	218507.81	210574.77	202855.10	209632.89

101	Carbohydrates	N-Acetylgalactosamine	171300.21	378693.20	135864.40	111251.45
102	Carbohydrates	Sucrose	104161.42	46264.46	80748.58	60093.39
103	Carbohydrates	Beta-D-Glucopyranuronic acid	35538.08	38214.60	25817.77	19610.03
104	Benzenoids	Aniline	16622218.25	16251895.10	16296956.05	16528009.79
105	Benzenoids	Cinnamic acid	2558297.72	2398866.62	2141064.23	2283136.39
106	Benzenoids	Cuminaldehyde	1440821.51	1393850.08	1398582.31	1403745.64
107	Benzenoids	4-Nitrophenol	492057.27	492147.59	489912.23	483611.02
108	Benzenoids	Estazolam	474504.46	481279.98	473280.79	514367.52
109	Benzenoids	Alpha-Methylstyrene	440384.73	488141.39	468711.92	456236.14
110	Benzenoids	6-pentadecyl Salicylic Acid	343519.10	564056.90	317949.28	348054.86
111	Benzenoids	Monoethylhexyl phthalic acid	152653.59	164697.18	153994.55	129357.51
112	Benzenoids	N-(2-Phenylethyl)-acetamide	83797.76	84291.87	85625.11	88547.59
113	Benzenoids	Zeranol	79095.33	77304.33	77570.45	65144.65
114	Benzenoids	Umbelliferone	84811.46	153674.96	170374.20	196411.54
115	Benzenoids	4-Hydroxycinnamoylmethane	52883.25	55400.85	47249.90	33738.04
116	Benzenoids	4-Hydroxybenzaldehyde	38770.05	31685.59	40007.66	43870.42
117	Benzenoids	Nirvanol	47511.10	25425.70	58869.36	53218.11

118	Benzenoids	Hydroxyphenyllactic acid	14290.97	8890.34	16295.52	14625.21
119	Benzenoids	4-Hydroxyaminoquinoline N-oxide	11569.15	17474.95	16218.60	29630.37
120	Benzenoids	P-Tolualdehyde	6741.61	5048.97	12855.21	18809.10
121	Organic nitrogen compounds	Edetate	36153107.25	35437433.28	32958546.03	35731274.68
122	Organic nitrogen compounds	3-Buten-1-amine	2599250.35	2557301.10	2568668.19	2641380.99
123	Organic nitrogen compounds	3-Pyridinaldehyde	631351.26	631849.83	634374.00	665351.36
124	Organic nitrogen compounds	Phosphocholine	591560.51	649728.43	647914.09	677766.58
125	Organic nitrogen compounds	2-Cyanopyridine	489497.07	487366.44	493550.79	516457.31
126	Organic nitrogen compounds	Dihydrozeatin	473537.84	466864.37	474794.04	494203.72
127	Organic nitrogen compounds	Trolamine	418167.25	394273.74	388127.28	373972.78
128	Organic nitrogen compounds	Trimethylamine N-oxide	173676.09	176340.03	193940.56	266781.45
129	Organic nitrogen compounds	Caldine	85248.83	74284.92	58237.84	65645.92
130	Organic nitrogen compounds	Ethyl carbamate	139859.65	52963.75	71360.49	56336.49
131	Organic nitrogen compounds	MEMANTINE	37356.63	40529.56	38572.20	36376.35
132	Organic nitrogen compounds	Isoxanthopterin	22817.11	16190.59	12689.70	5251.96
133	Organic nitrogen compounds	N,N-Dimethylaniline	21190.19	21874.40	22542.46	23147.68
134	Organic nitrogen compounds	Coniine	5010478.16	4970252.52	5012283.64	5190994.37

135	Organic nitrogen compounds	Robustine	850926.79	816355.15	832020.43	837841.70
136	Hormones and others	Acetylcholine	125180.20	134713.78	97290.06	143694.14
137	Hormones and others	Serotonin	45219.63	42645.59	35591.15	26440.55
138	Hormones and others	Crustecdysone	13862.88	123731.08	24980.06	57870.65
139	Hormones and others	2-Methylpropanal O-methyloxime	6355096.69	6358095.63	6405967.44	6623224.68
140	Hormones and others	17beta-Hydroxy-2alpha-(methoxymethyl)-17-methyl-5alpha-androstan-3-one	206725.85	317217.91	186618.00	199004.42
141	Hormones and others	Cis-4-Carboxymethylenebut-2-en-4-olide	124297.22	122552.86	127154.48	123615.05
142	Hormones and others	Phosphoric acid	51224.64	52931.43	54190.11	52997.99

Supplementary Table 2. List of primers used in this paper

Genes	Primers name	Sequence (5′-3′)	Amplicon size (bp)
**Primers for 16S rRNA sequencing**			
Bacterial 16S rRNA (V3-V4 region)	338F	ACTCCTACGGGAGGCAGCAG	469bp
	806R	GGACTACHVGGGTWTCTAAT	
**Primers for qPCR**			
Bacterial 16S rRNA	q-16S-891F	TGGAGCATGTGGTTTAATTCGA	113bp
	q-16S-1003R	TGCGGGACTTAACCCAACA	
Vibrio specific 16S rRNA gene fragment	q-*Vibrio*-F	GGCGTAAAGCGCATGCAGGT	205 bp
	q-*Vibrio*-R	GAAATTCTACCCCCCTCTACAG	
Shewanella specific 16S rRNA gene fragment	q-She-640F	RACTAGAGTCTTGTAGAGG	175 bp
	q-She-815R	AAGDYACCAAAYTCCGAGTA	
Staphylococcus aureus (*rpoB*)	q-Sa-F	CTAAGCACAGAGGTCGT	298bp
	q-Sa-R	ACGGCATCCTCATAGT	
purine nucleoside phosphorylase (PNP) (Genbank ID: XM_027371182.1)	q-PNP-F	CGTGGAGCAGGTCATCATCA	184 bp
	q-PNP-R	CTTGTCGTAGGCGTTGTTCAT	
nicotinamide phosphoribosyltransferase (NAMPT) (Genbank ID: XM_027381131.1)	q- NAMPT-F	CACCACCTTCAGTATCCACCT	162 bp
	q- NAMPT-R	CTCCTTCGCCTCCTGTATCAT	
nicotinamide riboside kinase 1 (NRK1) (Genbank ID: XM_027351750.1)	q- NRK1-F	CGTGGATGTCGCCAAGATG	260 bp
	q- NRK1-R	TCGTGAGCGTGAGGAAGAA	
	q- NRK2-F	TGGTGGCAAGTCTACCTTAACA	189 bp

nicotinamide riboside kinase 2 (NRK2) (Genbank ID: XM_027365064.1)	q- NRK2-R	AGCACATCCTTCTCCATTCTCT	
nicotinamide acid mononucleotide adenylyltransferase 1 (NMNAT) (Genbank ID: XM_027362542.1)	q- NMNAT-F	GTGGACTCTGTGGTCAATGGA	232 bp
	q- NMNAT-R	TGCTGCCTTCTCGTGTTATCA	
ectonucleotide pyrophosphatase family member 1 (ENPP1) (XM_027358130.1)	q- ENPP1-F	GTGGTGCTGGTGGTAATC	245 bp
	q- ENPP1-R	GTGACGATAGTGTAGTGGTT	
NAD-dependent protein deacylase Sirt4 (SIRT4) (Genbank ID: XM_027354144)	q- SIRT4 -F	GAGCAGGTATATCAACAGAGA	209 bp
	q- SIRT4-R	CCATTCAGCAAGTGTCAGA	
NAD-dependent protein deacetylase Sirt6 (SIRT6) (Genbank ID: XM_027372939.1)	q- SIRT6 -F	GGTAAGGAAGAGGTTGATGA	233 bp
	q- SIRT6-R	GTGGCATTGACGACAGAT	
**Primer for real-time RT-PCR**			
V. parahaemolyticus (*tlh*)	Vp-F	GATTTGGCGAACGAGAAC	695bp
	Vp-R	CGTCTCGAACAAGGCG	
V. harveyi (*vhh*)	Vh-F	CTTCACGCTTGATGGCTACTG	253bp
	Vh-R	GTCACCCAATGCTACGACCT	
V. parahaemolyticus (*pirB*)	PirB-F	GTGGGCTGATAACGACTC	187bp
	PirB-R	ACCAACAGCAGGTGAATA	
**Primers for dsRNA templates amplification**			
Enhanced green fluorescent protein (GFP) (Genbank ID: U55762.1)	dsGFP-F	CGTAAACGGCCACAAGTT	429bp
	dsGFP-R	TTCACCTTGATGCCGTTC	
	dsGFP-T7F	GGATCCTAATACGACTCACTATAGGCGTAAACG GCCACAAGTT	

	dsGFP-T7R	GGATCCTAATACGACTCACTATAGGTTCACCTTG ATGCCGTTC	
Hemocyanin (HMC) (Genbank ID: XM_027383261.1)	ds*Lv*HMC-F	GTCCTCATCCACTGCAAA	449bp
	ds*Lv*HMC-R	TTGGACAGACGTTCAGCA	
	dsLvHMC-T7F	GGATCCTAATACGACTCACTATAGGGTCCTCATC CACTGCAAA	
	dsLvHMC-T7R	GGATCCTAATACGACTCACTATAGGTTGGACAG ACGTTCAGCA	
